# Dose–Effect Relationship of the Immunotoxicity, Neurotoxicity, Gastrointestinal Toxicity, and Hepatotoxicity of the Maillard Reaction Product 2-Acetylfuran

**DOI:** 10.3390/foods15030432

**Published:** 2026-01-24

**Authors:** Qiaosi Wei, Xiangxin Wang, Qingxue Chen, Shubo Luo, Dongying Cui, Sinan Mu, Jufang Li, Qinggang Xie, Yajun Xu

**Affiliations:** 1 Department of Nutrition and Food Hygiene, School of Public Health, Peking University, No. 38 Xueyuan Road, Haidian District, Beijing 100083, China; weiqiaosi@feihe.com; 2 Heilongjiang Feihe Dairy Co., Ltd., C-16, 10A Jiuxianqiao Rd., Chaoyang, Beijing 100015, China; wangxiangxin@feihe.com (X.W.); luoshubo@feihe.com (S.L.); cuidongying@feihe.com (D.C.); musinan@feihe.com (S.M.); lijufang@feihe.com (J.L.); xieqinggang@feihe.com (Q.X.); 3 PKUHSC-China Feihe Joint Research Institute of Nutrition and Healthy Lifespan Development, Xueyuan Road 38, Haidian, Beijing 100083, China; 4 Food College, Northeast Agricultural University, Harbin 150030, China; snoopycqx@163.com; 5 Beijing Key Laboratory of Toxicological Research and Risk Assessment for Food Safety, Peking University, No. 38 Xueyuan Road, Beijing 100083, China

**Keywords:** 2-acetylfuran, zebrafish, immunotoxicity, hepatotoxicity

## Abstract

2-acetylfuran is a product of the Maillard reaction and is widely found, especially in heat-processed foods such as grain products, baked goods, and dairy products. Although 2-acetylfuran contributes to flavor, high concentrations may be toxic. Its target organs and dose–response relationships remain poorly characterized. In this study, transgenic zebrafish with fluorescently labeled immune and neural systems were used to assess the effects of 2-acetylfuran on immune and neural development. Wild-type zebrafish were employed to assess the toxicity of 2-acetylfuran on locomotor ability, gastrointestinal development, and liver function. The maximum non-lethal concentration (MNLC) and the 10% lethal concentration (LC_10_) for zebrafish embryos were 0.844 and 0.889 μL/mL, respectively. Regarding immunotoxicity, at concentrations of 0.281, 0.844, and 0.889 μL/mL, 2-acetylfuran significantly reduced the numbers of neutrophils, T cells, and macrophages. Regarding locomotor and neurotoxicity, motor speed and total locomotor distance were significantly reduced at 0.844 and 0.889 μL/mL. These findings were consistent with neurodevelopmental assessments, in which 0.844 μL/mL 2-acetylfuran resulted in a significant increase in apoptotic cells in the central nervous system and markedly shortened peripheral motor nerve lengths. Regarding gastrointestinal toxicity, 0.844 and 0.889 μL/mL 2-acetylfuran significantly reduced the gastrointestinal area, while neutrophil counts showed no significant changes, suggesting a relatively mild effect on the gastrointestinal tract. Regarding hepatic toxicity, all tested concentrations of 2-acetylfuran primarily increased the delayed yolk sac absorption area. Furthermore, at 0.844 μL/mL, histological examination revealed hepatic pathological changes characterized by hepatocyte nuclear swelling, vacuolar degeneration, and hepatocyte necrosis. In summary, this study reveals the multi-organ toxicity profile of 2-acetylfuran in the zebrafish model, with particularly high sensitivity in the immune system and liver. This research provides theoretical support for risk assessment and process control of 2-acetylfuran in foods.

## 1. Introduction

Thermal processing is widely applied in the production and storage of foods such as grain products, baked goods, coffee beverages, and infant formula. Although thermal processing improves product sensory quality and microbial safety, it simultaneously promotes the formation of various Maillard reaction products (MRPs) [1,2]. Among these products, volatile small molecules, such as furans and their derivatives, are considered major process contaminants in heat-processed foods that may pose potential health risks [3]. Epidemiological studies and animal experiments suggest that certain furan compounds may cause hepatotoxicity, neurotoxicity, and carcinogenicity. Consequently, increasing attention has been directed toward the levels of these compounds in food and feed, as well as their associated health effects [4].

2-acetylfuran is a typical furan-type Maillard product formed through dehydration, rearrangement, and cleavage reactions between reducing sugars and amino acids at elevated temperatures [5]. It serves as a food flavoring agent and occurs naturally in baked grains, roasted oils, and caramelized dairy products. Other Maillard reaction products with structural or biosynthetic relevance to 2-acetylfuran, such as 5-hydroxymethylfurfural (5-HMF), 2-furfural, and glyoxal, have been shown to cause developmental, neurotoxic, and hepatotoxic effects in zebrafish, rodents, and cellular models. 5-HMF and 2-furfural can induce developmental delays, impaired bone mineralization, and pathological changes in the liver and central nervous system of zebrafish [6,7]. Glyoxal has also been reported to impair organ development and trigger oxidative stress and inflammatory responses [8]. Collectively, these findings suggest that Maillard reaction products, despite their relatively low levels in heat-processed foods, may still exert cumulative health effects following chronic or early-life exposure. Currently, systematic toxicological data on 2-acetylfuran remain extremely limited. A 90-day evaluation in rats confirmed that 22.6 mg/kg body weight daily is a safe dose of 2-acetylfuran [9]. Furthermore, the EFSA Animal Feed Additives and Products or Substances Group has reported safe doses of 2-acetylfuran in several species, including cats (2 mg/kg body weight), dogs (11.9 mg/kg body weight), and ornamental fish (44.2 mg/kg body weight) [10]. Based on these findings, the group states that the use of 2-acetylfuran in animal feed at doses ≤ 0.5 mg/kg is safe in all animal diets. These findings provide a safe range for 2-acetylfuran exposure, but toxicity from high-concentration exposures has not yet been reported. In particular, there is a lack of dose–response data on the effects of 2-acetylfuran on specific organs, such as the immune system, nervous system, gastrointestinal tract, and liver.

Zebrafish embryos and larvae possess transparent bodies and a well-characterized developmental timeline and exhibit high genetic and physiological similarity to humans. These features have established zebrafish as a key vertebrate model for evaluating the toxicity of environmental pollutants and foodborne hazards [11,12]. In addition, their immune, nervous, gastrointestinal, and hepatic systems share substantial structural and functional homology with those of mammals, enabling reliable assessment of developmental and functional toxicity across multiple organs. Transgenic strains allow visual tracking of target cells, such as immune cells and motor neurons [13]. As a result, zebrafish are widely used in toxicological assessments to elucidate the mechanisms of neurotoxicity, hepatotoxicity, and immunotoxicity caused by metals, pesticides, plastic additives, and novel food contaminants [14,15]. Compared with traditional rodent models, zebrafish are better suited for early developmental exposure studies and high-throughput screening. Furthermore, zebrafish can generate a comprehensive toxicity profile using multidimensional endpoints, including locomotor behavior, immune cell fluorescence, and organ morphology.

In this context, zebrafish were used as a model organism to determine the maximum non-lethal concentration (MNLC) and the 10% lethal concentration (LC_10_) of 2-acetylfuran using concentration–lethality curves. Based on these findings, four exposure doses (1/9 of MNLC, 1/3 of MNLC, MNLC, and LC_10_) were chosen to systematically evaluate the effects of 2-acetylfuran on zebrafish immune systems, locomotor behavior, neural development, gastrointestinal development, and liver function. Overall, this study aims to elucidate the multi-organ toxicity profile of 2-acetylfuran and clarify its dose–response relationships.

## 2. Materials and Methods

### 2.1. Zebrafish Husbandry

Wild-type AB zebrafish and transgenic lines were obtained from Hunter Biotechnology Inc. (Hangzhou, China). All fish were maintained at 28 °C in recirculating aquaria with prepared culture water consisting of reverse osmosis water, supplemented with 200 mg/L of instant sea salt, with conductivity adjusted to 450–550 µS/cm, pH maintained between 6.5 and 8.5, and a total hardness of 50–100 mg/L CaCO_3_. Animal use was approved by the Institutional Animal Care and Use Committee (IACUC-2023-6809-01). All procedures complied with the institutional license for experimental animals (SYXK (Zhejiang) 2022-0004) and adhered to AAALAC accreditation requirements (accreditation No. 001458).

### 2.2. Determination of MNLC and LC_10_ for 2-Acetylfuran

The acute lethality of 2-acetylfuran (purity of 99%, Merck KGaA, Darmstadt, Germany) was first assessed in 2-day-post-fertilization (dpf) wild-type AB zebrafish larvae. Larvae were exposed for 3 days to a series of nominal 2-acetylfuran concentrations (0.062, 0.125, 0.250, 0.500, and 1.00 µL/mL). A normal control (NC) and a vehicle control containing 1% DMSO were included in parallel. Simultaneously, all concentrations of 2-acetylfuran were dissolved in 1% DMSO. Zebrafish were reared in 6-well plates with 30 zebrafish per well. To each well, 3 mL of aqueous solution containing or without 2-acetylfuran (dissolved in 1% DMSO) was added. Mortality in each group was recorded daily, and dead larvae were promptly removed. Origin 8.0 software was used to fit the concentration–mortality ratio of 2-acetylfuran, and a nonlinear fitting was employed to generate the optimal concentration–mortality effect curve for 2-acetylfuran. The MNLC and LC_10_ of 2-acetylfuran were recorded using the 1% and 10% mortality rates from the curve, respectively. These values were then used to define a subtoxic exposure concentration set (1/9 of MNLC, 1/3 of MNLC, MNLC, and LC_10_) for subsequent toxicity assessments.

### 2.3. Immunotoxicity Assessment

Immunotoxic effects of 2-acetylfuran were examined using three transgenic lines that label distinct immune cell populations: Tg (mpx: EGFP) for neutrophils, Tg (mpeg1: EGFP) for macrophages, and Tg (rag2: DsRed) for T cells in the thymus. The three transgenic zebrafish strains were provided by Hunter Biotechnology Inc. (Hangzhou, China). At 3 dpf, larvae were randomly assigned to six treatment groups (*n* = 30 per group): the NC, 1% DMSO, and four 2-acetylfuran exposure concentrations corresponding to 1/9 of the MNLC (0.094 µL/mL), 1/3 of the MNLC (0.281 µL/mL), the MNLC (0.844 µL/mL), and LC_10_ (0.889 µL/mL). Exposure was carried out for 2 days at 28 °C. After exposure,10 larvae from each group were randomly selected and mounted for imaging under a fluorescence microscope. Images of the caudal vein region were acquired, and the numbers and fluorescence intensities of neutrophils, macrophages, and T cells were quantified using the NIS-Elements D 3.20 advanced imaging software (Nikon, Tokyo, Japan). Changes in immune cell abundance in the tail vasculature were considered indicators of immunotoxicity.

### 2.4. Behavioral Analysis

Locomotor activity was assessed in wild-type AB larvae at 4 dfp to evaluate 2-acetylfuran-induced motor function alterations. The same six exposure groups and sample sizes as described in Section 2.3 were employed (*n* = 30 per group). Larvae were exposed to the respective concentrations for 1 day at 28 °C. After exposure, 10 larvae per group were randomly selected and individually transferred into the wells of a behavior analysis system (V3.11, ViewPoint Life Sciences, Civrieux, France). Spontaneous locomotion was recorded for 20 min. The total distance traveled was calculated over 20 min, and average swimming speed per minute was further analyzed during a light–dark challenge paradigm consisting of two cycles of 5 min dark and 5 min light phases (20 min in total).

### 2.5. Apoptotic Cell Detection in the Central Nervous System

Wild-type AB larvae at 6 dpf were used to investigate whether 2-acetylfuran induces apoptosis in the central nervous system (CNS). Larvae were allocated to the same six exposure groups described in Section 2.3 (*n* = 30 per group) and treated with the indicated concentrations for 2 days at 28 °C. After exposure, the larvae were stained with acridine orange (AO) in the dark for 30 min. Following staining, the larvae were rinsed three times with fresh culture water to remove excess dye. Ten larvae from each group were then randomly selected for fluorescence microscopy, and AO fluorescence in the CNS region was quantified as an indicator of apoptotic cell burden.

### 2.6. Assessment of Peripheral Motor Neuron Length

The impact of 2-acetylfuran on peripheral motor neuron development was evaluated using 6 dpf Tg (hb9: EGFP) larvae, in which motor neurons express green fluorescence. Larvae were assigned to the same treatment groups as described in Section 2.3 (*n* = 30 per group) and exposed to the respective 2-acetylfuran concentrations for 3 days at 28 °C. Following exposure, 10 larvae per group were randomly selected for the fluorescence microscope. The lengths of peripheral motor nerves within the region encompassing three somites dorsal to the cloaca were measured using image analysis software. Mean motor nerve length per fish was calculated for each group and used as an endpoint for neurodevelopmental toxicity.

### 2.7. Gastrointestinal Area Measurement

To assess gastrointestinal (GI) development, 3 dpf wild-type AB larvae were exposed to 2-acetylfuran. The grouping strategy and exposure concentrations were identical to those described in Section 2.3, with 30 larvae per group. After 2 days of exposure, 10 larvae from each group were randomly selected and imaged under a dissecting microscope (SZX7, OLYMPUS, Tokyo, Japan). The GI tract area was outlined and quantified using image analysis software, and the relative GI area was compared across groups as an indicator of developmental delay or growth impairment in the digestive system.

### 2.8. Quantification of Neutrophils in the Gastrointestinal Tract

Gastrointestinal (GI) inflammation was further evaluated using 3 dpf Tg (mpx: EGFP) larvae, in which neutrophils are fluorescently labeled. Larvae were assigned to the same six treatment groups as described in Section 2.3 (30 larvae per group) and exposed to the respective concentrations of 2-acetylfuran for 2 days. Following exposure, the larvae were imaged using a fluorescence microscope. The number of neutrophils within the defined GI tract region was counted and normalized to the GI area to obtain neutrophil density (cells per unit area). This parameter was used to evaluate GI inflammatory responses induced by 2-acetylfuran.

### 2.9. Hepatotoxicity Evaluation

Hepatotoxicity was examined in wild-type AB larvae using the same six treatment groups and group sizes as in Section 2.3. Larvae were exposed to 2-acetylfuran for 2 days. After exposure, 10 larvae per group were randomly selected and observed under a dissecting microscope. Liver morphology was documented, and quantitative analysis of liver area, mean liver brightness (as a proxy for hepatocellular integrity), and yolk sac residual area was performed using image analysis software. These parameters were collectively used to characterize hepatotoxic changes.

### 2.10. Histopathological Analysis (H&E Staining)

To confirm liver injury histopathologically, larvae from the hepatotoxicity experiments (Section 2.9) were collected for hematoxylin and eosin (H&E) staining. Zebrafish were fixed in 4% paraformaldehyde and processed using a standard histological workflow, including graded dehydration, paraffin embedding, sectioning, and H&E staining. Liver sections were examined under a light microscope, and histopathological alterations were evaluated to corroborate the quantitative liver toxicity endpoints.

### 2.11. Statistical Analysis

All quantitative data are presented as the mean ± standard deviation (SD), with *n* = 3–10 per group depending on the specific assay. One-way analysis of variance (ANOVA) was used to compare differences among groups, followed by Tukey’s post hoc test for pairwise comparisons. Data processing was performed using SPSS version 26.0 (IBM SPSS Inc., New York, NY, USA). *p* values < 0.05 were considered statistically significant, and differences are indicated by different letters. Furthermore, all fluorescence-related images acquired in this study were not edited or manipulated outside of standard processing procedures. Because the images were acquired while the zebrafish were alive, all images were region-specific, and complete images were not collected.

## 3. Results

### 3.1. MNLC and LC_10_ Determination and Acute Toxicity Assessment of 2-Acetylfuran

**Figure 1:**
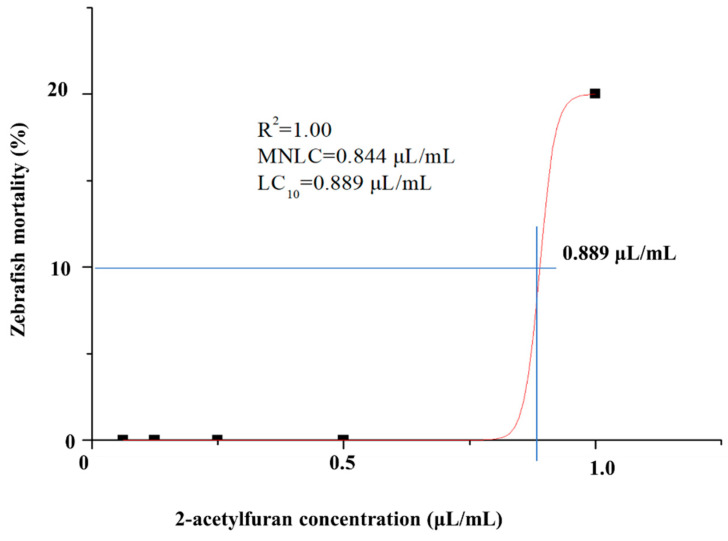
A fitted concentration–response curve is plotted to show the relationship between 2-acetylfuran concentration and zebrafish mortality. The horizontal axis represents 2-acetylfuran concentration, whereas the vertical axis represents mortality rate. The intersection point of the blue line indicates LC_10_. Data are expressed as the mean ± SD (*n* = 30).

Mortality was assessed in zebrafish larvae exposed to 2-acetylfuran at 0.062, 0.125, 0.250, 0.500, and 1.000 μL/mL. The results in Table 1 indicate that zebrafish mortality increased with rising 2-acetylfuran concentrations. Initial lethality occurred at 0.250 μL/mL, resulting in 3% mortality, and complete mortality was observed at 1.000 μL/mL. The fitted curve yielded LC_10_ and MNLC values of 0.889 μL/mL and 0.844 μL/mL, respectively (Figure 1). Next, the effects of 1/9 of the MNLC (0.094 μL/mL), 1/3 of the MNLC (0.281 μL/mL), the MNLC (0.844 μL/mL), and LC_10_ (0.889 μL/mL) on the zebrafish’s immunity, locomotor ability, nervous system, gastrointestinal tract, and liver were evaluated.

### 3.2. Immunotoxicity of 2-Acetylfuran

**Figure 2:**
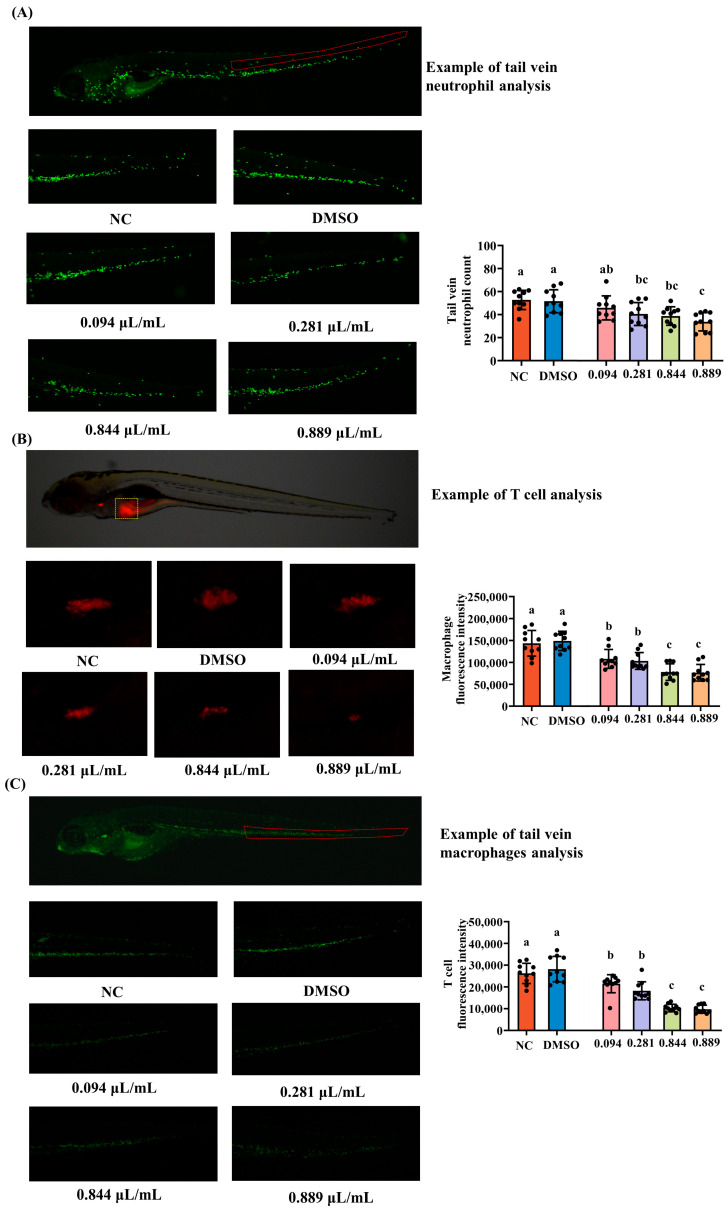
Effects of different concentrations of 2-acetylfuran on immune cells in zebrafish. (**A**) Fluorescence images of neutrophils (the area marked with red lines). (**B**) Fluorescence images of T cells (the area marked with yellow lines). (**C**) Fluorescence images of macrophages (the area marked with red lines). The quantitative fluorescence analysis of neutrophils, T cells, and macrophages is represented by a bar chart on the right side of the corresponding fluorescence image. Data are expressed as the mean ± SD (*n* = 10). *p* values < 0.05 were considered statistically significant, and differences are indicated by different letters.

Neutrophils, macrophages, and T cells are key innate and adaptive immune cells that contribute to inflammatory responses, phagocytosis, and immune regulation. To evaluate the immunotoxic effects of 2-acetylfuran, changes in the abundance of these cells were quantified in zebrafish larvae. To assess immunotoxicity, the effects of graded concentrations of 2-acetylfuran on zebrafish immune cells were quantified to establish concentration–response relationships. Representative fluorescence images of neutrophils, T cells, and macrophages are shown in Figure 2A–C. Overall, the fluorescence intensity of neutrophils (green), T cells (green), and macrophages (red) decreased with increasing concentrations of 2-acetylfuran. Subsequently, the fluorescence expression levels of neutrophils, T cells, and macrophages were quantified based on image annotations, as shown in Figure 2A–C. Compared to the NC, 2-acetylfuran concentrations of 0.281 μL/mL, 0.844 μL/mL, and 0.889 μL/mL significantly reduced neutrophil counts (*p* < 0.05), whereas the 0.094 μL/mL concentration showed no significant change (*p* > 0.05). Furthermore, maximum neutrophil toxicity was observed at a concentration of 0.889 μL/mL. In T cells and macrophages, all four concentrations of 2-acetylfuran significantly reduced cell counts compared to the NC (*p* < 0.05), with maximum toxicity observed at 0.844 μL/mL. These results indicate that 2-acetylfuran exhibited pronounced immunotoxicity, with T cell and macrophage toxicity evident even at 1/9 of the maximum non-lethal concentration (MNLC).

### 3.3. Effects of 2-Acetylfuran on Motor Function in Zebrafish

**Figure 3:**
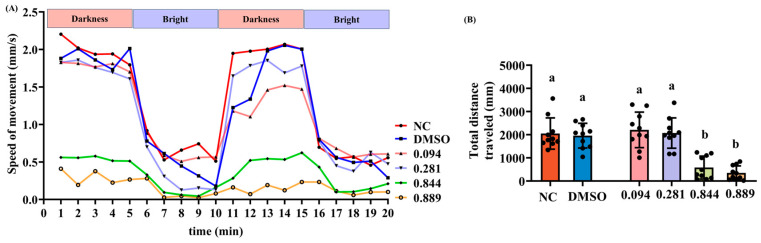
Effects of different concentrations of 2-acetylfuran on zebrafish locomotor activity. (**A**) Changes in zebrafish swimming speed during 20 min of light–dark cycles. (**B**) Total swimming distance of zebrafish over 20 min. Data are the expressed as the mean ± SD (*n* = 10). *p* values < 0.05 were considered statistically significant, and differences are indicated by different letters.

The locomotor activity of zebrafish larvae was assessed following exposure to graded concentrations of 2-acetylfuran. As shown in Figure 3A, zebrafish locomotion speeds were recorded over 20 min during alternating light–dark cycles. All groups moved faster in the dark environment, indicating zebrafish phototaxis toward darkness. During the first dark phase, zebrafish in the NC, DMSO, 0.094 μL/mL, and 0.281 μL/mL groups exhibited similar swimming speeds (~2.0 mm/s), while those in the 0.844 μL/mL and 0.889 μL/mL groups showed significantly reduced speeds (~0.5 mm/s). In the second dark phase, the NC and DMSO groups maintained their previous swimming speeds (~2.0 mm/s). However, the 0.094 μL/mL and 0.281 μL/mL groups showed a slight decrease in speed (~1.5 mm/s). The 0.844 μL/mL and 0.889 μL/mL groups still showed low swimming speeds (~0.5 mm/s). Across both light and dark periods, swimming speeds in the NC, DMSO, 0.094, and 0.281 μL/mL groups were consistently higher than those in the 0.844 and 0.889 μL/mL groups. Total swimming distance was then quantified, as shown in Figure 3B. Compared with that in the NC group, the total swimming distance was significantly reduced in the 0.844 μL/mL and 0.889 μL/mL groups (*p* < 0.05), while there was no significant change in the 0.094 μL/mL and 0.281 μL/mL groups (*p* > 0.05). Furthermore, no significant difference in total swimming distance was observed between the 0.094 μL/mL and 0.281 μL/mL groups (*p* > 0.05). In summary, these results indicate that 2-acetylfuran reduces locomotor ability, with its toxic dose corresponding to MNLC levels.

### 3.4. Neurotoxicity of 2-Acetylfuran

**Figure 4:**
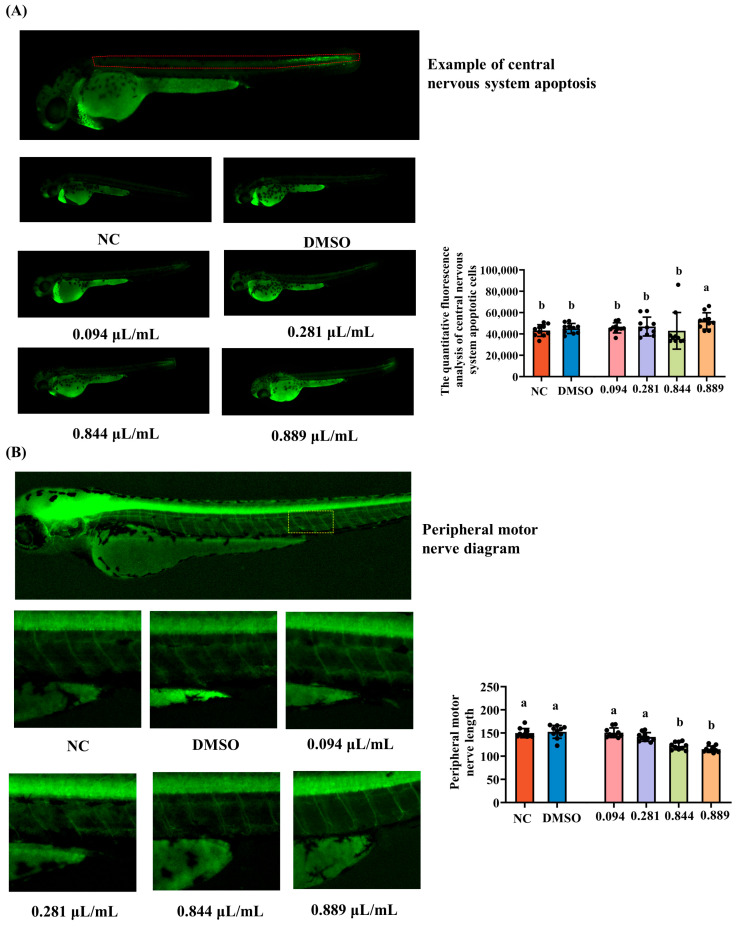
Effects of different concentrations of 2-acetylfuran on neurotoxicity in zebrafish. (**A**) Fluorescent images of central nervous system apoptosis (the area marked with red lines). (**B**) Fluorescent images of peripheral motor neuron length (the area marked with yellow lines). The quantitative fluorescence analysis of central nervous system apoptotic cells and peripheral motor neuron length is represented by a bar chart on the right side of the corresponding fluorescence image. Data are expressed as the mean ± SD (*n* = 10). *p* values < 0.05 were considered statistically significant, and differences are indicated by different letters.

The effects of different concentrations of 2-acetylfuran on apoptotic cells in the central nervous system (CNS) of zebrafish were evaluated. As shown in Figure 4A, CNS cells were distributed throughout the dorsal region of zebrafish, with lower fluorescence intensity in the NC and DMSO groups. The fluorescence intensity increased in a dose-dependent manner and was significantly enhanced at 0.889 μL/mL. This finding was confirmed by annotated fluorescence counts, which showed that the number of central nervous system apoptotic cells was significantly higher in the 0.889 μL/mL group compared to the NC group (*p* < 0.05), while no significant differences were observed between the other groups and the NC group (*p* > 0.05, Figure 4A). Peripheral motor nerves consist of motor neuron axons responsible for transmitting motor commands from the central nervous system to the muscles, serving as a direct quantitative indicator of neural development. Fluorescent images and fluorescence intensities of motor nerve lengths are shown in Figure 4B. Compared to that in the NC group, motor nerve length was significantly reduced in the 0.844 μL/mL and 0.889 μL/mL groups (*p* < 0.05), while no significant changes were observed at 0.094 or 0.281 μL/mL (*p* > 0.05). This finding correlates with changes in zebrafish locomotor ability, suggesting that motor neurons regulate locomotor function. Collectively, the increased CNS apoptosis, shortened motor axons, and impaired locomotion at concentrations near LC_10_ demonstrate apparent neurotoxic effects of 2-acetylfuran in zebrafish.

### 3.5. Gastrointestinal Toxicity of 2-Acetylfuran

**Figure 5:**
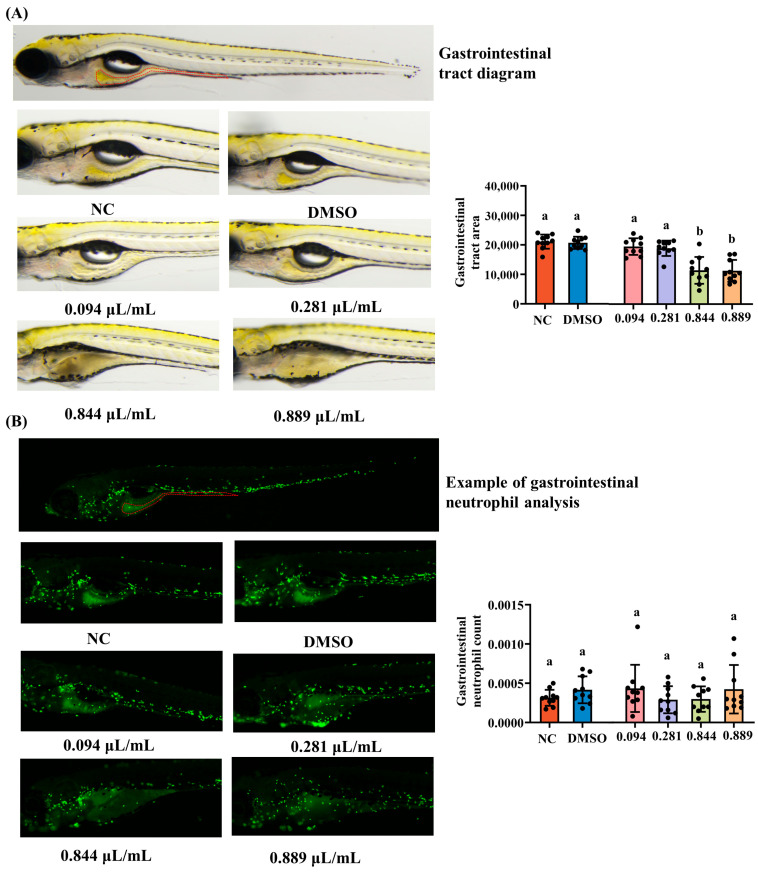
Effects of different concentrations of 2-acetylfuran on gastrointestinal toxicity in zebrafish. (**A**) Gastrointestinal dissection diagram (the area marked with red lines). (**B**) Fluorescent images of neutrophils in the gastrointestinal tract (the area marked with red lines). The results of quantitative fluorescence analysis of gastrointestinal tract area and gastrointestinal neutrophils are shown in bar charts on the right side of the corresponding images. Data are expressed as the mean ± SD (*n* = 10). *p* values < 0.05 were considered statistically significant, and differences are indicated by different letters.

The foregut, midgut, and hindgut of zebrafish exhibit high morphological and functional similarity to mammalian digestive tracts. Gastrointestinal morphology was examined using a dissecting microscope, as shown in Figure 5A. The intestinal tract remained morphologically normal in the NC, DMSO, 0.094 μL/mL, and 0.281 μL/mL groups but exhibited severe deformation at 0.844 and 0.889 μL/mL. Intestinal surface area was markedly reduced at 0.844 and 0.889 μL/mL compared with that in the NC group (*p* < 0.05), whereas lower concentrations showed no significant difference (*p* > 0.05). The zebrafish intestine possesses a mucosal barrier and immune cells that functionally parallel mammalian intestinal immunity. Neutrophils are widely distributed throughout the zebrafish gastrointestinal tract. In this study, transgenic zebrafish were used to label neutrophils with green fluorescence, enabling direct visualization of their distribution and recruitment within the gastrointestinal tract. As shown in Figure 5B, substantial green fluorescence was observed throughout the gastrointestinal tract segments of all groups. Quantitative fluorescence analysis further revealed no significant differences in neutrophil counts across gastrointestinal tract segments (*p* > 0.05).

### 3.6. Hepatotoxicity of 2-Acetylfuran

**Figure 6:**
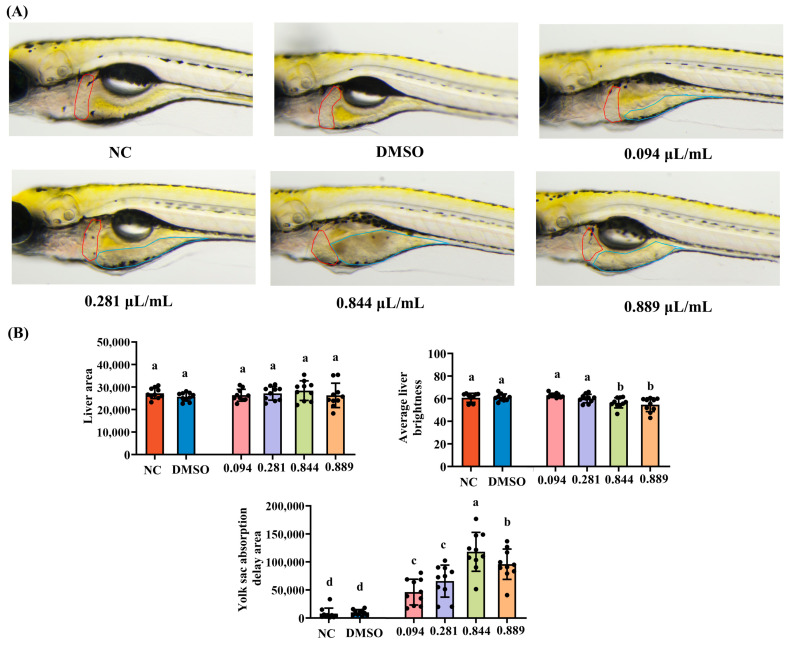

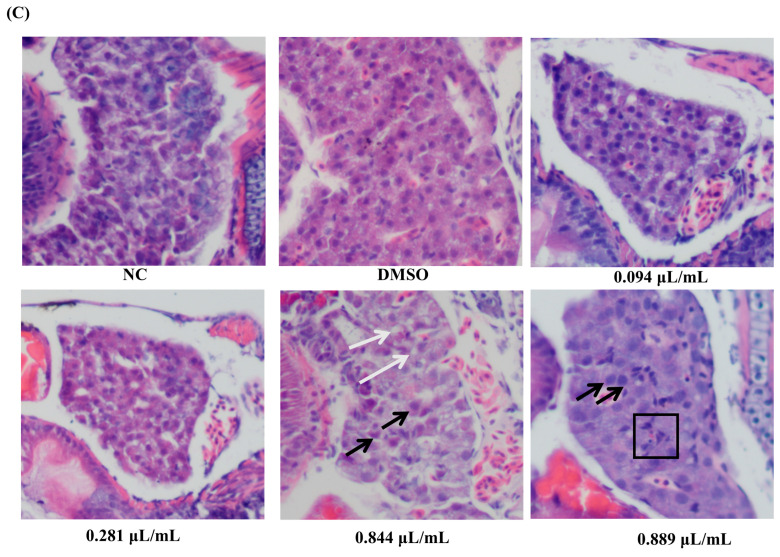
Effects of different concentrations of 2-acetylfuran on liver toxicity in zebrafish. (**A**) Liver dissection diagram: The red curve marks the location of the liver, and the blue curve marks the location of the yolk sac. (**B**) Liver area, liver brightness, and yolk sac absorption delay area. (**C**) H&E staining of liver tissue. In this diagram, black arrows represent cell nucleus enlargement, white arrows represent vacuolar degeneration, and black boxes represent necrotic foci. Data are expressed as the mean ± SD (*n* = 10). *p* values < 0.05 were considered statistically significant, and differences are indicated by different letters.

Zebrafish liver morphology, along with analyses of liver area and brightness, is presented in Figure 6A,B. Compared to the NC group, liver brightness was significantly reduced in the 0.844 μL/mL and 0.889 μL/mL groups (*p* < 0.05), while no significant changes were observed in the 0.094 μL/mL and 0.281 μL/mL groups (*p* > 0.05). Furthermore, no significant differences in liver area were observed between the groups (*p* > 0.05). The delayed yolk sac absorption area is commonly used as an indicator of early development and hepatic metabolic toxicity in zebrafish. During early development, the yolk sac provides energy and nutrients; an excessively high delayed absorption area indicates delayed liver development and lipid metabolism disorders. Our results showed that all tested concentrations of 2-acetylfuran significantly increased the delayed yolk sac absorption area (*p* < 0.05), demonstrating pronounced hepatic toxicity. Zebrafish liver tissue was then isolated and examined using HE staining. Hepatocytes in the NC and DMSO groups displayed abundant cytoplasm, large, round nuclei, clear and regularly arranged cell structures, and a small number of lipid droplets (Figure 6C). Following exposure to 0.844 μL/mL of 2-acetylfuran, zebrafish hepatocytes exhibited nuclear swelling, and vacuolar degeneration appeared in liver tissue. After exposure to 0.889 μL/mL of 2-acetylfuran, a distinct area of hepatocyte necrosis was visible, accompanied by hepatocyte nuclear swelling.

## 4. Discussion

2-acetylfuran is produced during the Maillard reaction, a process involving the dehydration, rearrangement, and cleavage of reducing sugars and amino acids. This process primarily occurs during the processing of protein- and carbohydrate-rich foods or feeds [5,16,17]. Furthermore, 2-acetylfuran is a commonly used flavoring additive in feeds, thereby exposing mammals or fish to its diet [10]. In recent years, the toxicity risks of Maillard reaction products such as 2-acetylfuran have received increasing attention. This study systematically examined the effects of 2-acetylfuran on immunity, locomotor behavior, neural and gastrointestinal development, and liver function at sublethal doses using a zebrafish multi-organ toxicity assessment system. Together with the determination of the MNLC and LC_10_, these analyses enabled delineation of the preliminary dose–response characteristics of 2-acetylfuran. The LC_10_ of 2-acetylfuran for zebrafish embryos was determined to be 0.889 μL/mL, while the MNLC was 0.844 μL/mL. Based on these values, concentrations of 1/9 of the MNLC, 1/3 of the MNLC, the MNLC, and LC_10_ were selected for sublethal toxicity assessment. One study reported 2-acetylfuran levels in 24 fruit- and meat-based canned infant food samples, all <6 ng/g [17]. This concentration is less than 1/9 of the MNLC in this study, reflecting that conventionally processed and normally stored products are generally not toxic due to 2-acetylfuran. Another study found 2-acetylfuran concentrations as high as 359.49 ng/g in oxidized fish oil [18]. This concentration is higher than 1/3 of the MNLC in this study. This suggests that the product may pose a risk of 2-acetylfuran toxicity under conditions of overprocessing or improper storage. In conclusion, these results indicate that the concentration tested in this study is of practical significance. A similar zebrafish toxicity assessment strategy based on the MNLC and LC_10_ has been widely applied in safety studies of Maillard reaction products, including 5-HMF, 2-furfural, and glyoxal [19,20]. Within the dose range that does not induce mass mortality, 2-acetylfuran can still cause significant functional or developmental damage to specific target organs, suggesting that reliance solely on traditional lethal endpoints may underestimate its actual health risks.

This study found that 2-acetylfuran exhibits differential sensitivity across various immune cell types with respect to immunotoxicity. Neutrophil counts decreased significantly at or above 1/3 of the MNLC, whereas T cells and macrophages exhibited marked reductions at doses as low as 1/9 of the MNLC. Neutrophils and macrophages are key effector cells in zebrafish’s innate immunity, participating in pathogen clearance, inflammatory responses, and tissue repair [21]. T cells are critical for adaptive immune development and the establishment of immune tolerance [22]. Previous studies have shown that various environmental pollutants and food-related small molecules can alter the abundance and distribution of neutrophils, macrophages, and thymic T cells in zebrafish at low doses [23,24]. The greater susceptibility of T cells and macrophages suggests that hematopoietic or lymphoid lineage cells may be more vulnerable to 2-acetylfuran than innate myeloid cells. This may be related to differences in proliferation and differentiation states, antioxidant capacity, and metabolic enzyme expression profiles between cell types. Previous studies indicate that 5-HMF disrupts immune cell homeostasis in zebrafish by inducing oxidative stress and affecting Nrf2 and downstream antioxidant gene expression [19]. Given its structural similarity to 5-HMF, 2-acetylfuran may undergo reactions analogous to those of 5-HMF (e.g., nucleophilic additions) and thereby induce oxidative stress and immune-cell dysfunction [18,25]. In summary, the immune system, particularly during early development, exhibits high sensitivity to small-molecule hazards. Even low-dose exposure to 2-acetylfuran may impair innate and adaptive immune function, warranting special attention for infants and children whose immune systems are not fully mature.

Behavioral results indicated that exposure to 2-acetylfuran at the MNLC and LC_10_ significantly reduced both locomotor speed and total distance traveled in zebrafish during light–dark cycle stimulation, resulting in markedly decreased activity. No significant effects were observed at the two lower concentrations (0.094 and 0.281 μL/mL). This dose-dependent change correlated strongly with reduced peripheral motor neuron axon length and increased central nervous system apoptosis, suggesting that the locomotor toxicity of 2-acetylfuran is closely associated with neurodevelopmental impairments [26]. Normal extension of motor neuron axons is critical for neuromuscular junction formation and effective locomotor control in zebrafish [27]. Reduced axon length often indicates impaired motor neuron development, leading to diminished locomotor capacity [12]. Maillard reaction products, environmental pollutants, and drug metabolites have been reported to exhibit neurotoxicity and motor toxicity similar to the neurotoxicity of 2-acetylfuran [19,28]. These studies generally agree that oxidative stress, mitochondrial dysfunction, and inflammatory responses are key mechanisms that induce neuronal apoptosis and impair axonal development. Therefore, 2-acetylfuran likely exerts its effects through similar pathways. Furthermore, the significant alterations in neurotoxic endpoints were observed primarily at the MNLC and LC_10_, indicating that the nervous system is less sensitive to 2-acetylfuran exposure than the immune system. This finding suggests differential susceptibility among organs to 2-acetylfuran exposure.

The effects of 2-acetylfuran on gastrointestinal development and mucosal immunity in zebrafish were subsequently evaluated. Results showed a significant reduction in gastrointestinal surface area of zebrafish only at the MNLC and LC_10_. Concurrently, the number and density of neutrophils in the gastrointestinal region did not differ significantly among the dose groups. In summary, these findings suggest that 2-acetylfuran exhibits low gastrointestinal toxicity, with reduced gastrointestinal surface area observed only at the MNLC. However, zebrafish are stomachless teleosts; their foregut, midgut, and hindgut exhibit structural and functional similarities to those of mammals. Their transparent body walls facilitate whole-organ observation, making them widely used to assess the effects of foodborne hazards on digestive system development and mucosal immunity [29]. In most intestinal toxicity models, significant intestinal inflammation is typically accompanied by massive neutrophil recruitment and destruction of mucosal structures [22]. In contrast, exposure to 2-acetylfuran reduced intestinal surface area, while neutrophil counts remained broadly stable. This suggests that its primary effect on the intestine may be developmental delay or growth restriction, rather than typical inflammatory damage.

Liver-related endpoints were more sensitive to 2-acetylfuran than gastrointestinal endpoints. This study revealed a significant reduction in liver fluorescence brightness at the MNLC and LC_10_ in zebrafish, suggesting alterations in hepatocellular refractive substances such as lipids or glycogen [30]. Specifically, the delayed yolk sac absorption area increased significantly at all tested concentrations. The yolk sac is the primary energy and nutrient source during early zebrafish development, and its absorption depends on normal liver and metabolic function. Delayed yolk sac absorption is a sensitive indicator of liver developmental delay or lipid metabolism disorders [31]. Accordingly, these results indicate that even at the lowest concentration (1/9 of MNLC), 2-acetylfuran significantly interferes with nutrient mobilization and liver-related metabolic functions in zebrafish. Histological analysis further confirmed the hepatotoxicity of 2-acetylfuran: in the 0.844 μL/mL treatment group, hepatocyte nuclear swelling, cytoplasmic vacuolation, and focal necrosis were clearly visible. These pathological alterations closely resemble the hepatic cell degeneration, steatosis, and necrosis phenotypes induced by 5-HMF and certain furan compounds in zebrafish and rodent models [19,20]. Together, these findings suggest that 2-acetylfuran may disrupt hepatic cell function by interfering with lipid metabolism, inducing oxidative stress, and causing mitochondrial damage.

## 5. Conclusions

Overall, this study represented the first multidimensional systematic evaluation of 2-acetylfuran toxicity and its dose–response relationships in zebrafish across immune, motor, neurodevelopmental, gastrointestinal, and hepatic systems. It showed that 2-acetylfuran induces immunosuppression and hepatic metabolic disturbances even at sublethal exposures, whereas higher sublethal concentrations elicit pronounced neurobehavioral toxicity and impair gastrointestinal development. These findings provided experimental evidence to inform food-safety assessments of 2-acetylfuran and related furan-type Maillard reaction products and offered valuable guidance for developing process-optimization and risk-management strategies.

## Figures and Tables

**Table 1:** 2-acetylfuran lethality (*n* = 30).

Concentration (μL/mL)	Death Toll	Mortality Rate (%)
0.062	0	0
0.125	0	0
0.250	1	3
0.500	5	17
1.000	30	100

## Data Availability

The original contributions presented in this study are included in the article. Further inquiries can be directed to the corresponding author.
